# Laparoscopic Cholecystectomy in a Morbidly Obese Patient With Situs Inversus Totalis: A Case Report

**DOI:** 10.7759/cureus.32304

**Published:** 2022-12-07

**Authors:** Vugar Suleimanov, Hadi Al Asker, Kawther Al Hawaj, Irfan W Alhashim, Fatemah N Al Rebh

**Affiliations:** 1 Surgery, Jubail General Hospital, Jubail, SAU; 2 General Surgery, Jubail General Hospital, Jubail, SAU

**Keywords:** american mirror technique, laparoscopic cholecystectomy (lc), symptomatic cholelithiasis, morbid obesity, situs inversus totalis (sit)

## Abstract

Situs inversus totalis (SIT) is a rare congenital condition characterized by a mirror-image transposition of both the abdominal and the thoracic organs. Due to the reversal of organs, laparoscopic cholecystectomy (LC) poses a significant challenge in patients with SIT. After the first reported case of LC in a patient with SIT in 1991, 120 more such reports have been published in the literature, but very few of them were carried out on morbidly obese patients. We report a morbidly obese patient, a known case of SIT, who presented with persistent biliary colic and underwent successful laparoscopic cholecystectomy in our institution. At surgery we used reverse Trendelenburg position with left tilt up and mirror-image of usual laparoscopic cholecystectomy port sites for the procedure. The procedure proved to be challenging, both due to the morbid obesity of the patient and the reversal of organs, which affected orientation and dexterity. A successful outcome has been reported in all the cases before us as well as our case, but it is noteworthy to mention that such cases must be performed by well-trained laparoscopic surgeons with impeccable manual dexterity who must take extreme care to avoid iatrogenic injuries.

## Introduction

Situs inversus is a rare autosomal recessive congenital disorder [1]. It includes a variety of conditions, where either abdominal, thoracic, or both organs are reversed. Situs inversus may be complete (totalis) or partial (partialis). In the case of situs inversus totalis (SIT) both thoracic and abdominal organs are reversed, whereas in the case of situs inversus partialis either thoracic or abdominal organs are reversed [2].

Situs inversus was first reported by Fabrcius in the beginning of the 17th century [3]. The Incidence is reported to be 1 in 6000 to 1 in 20000 live births. Several conditions have been associated with situs inversus, Kartagener’s syndrome being one of them, when the patient with SIT suffers from bronchiectasis and sinusitis. Some cardiac anomalies have been reported to be associated with SIT as well [4].

Situs inverses totalis is not known to increase the risk of gallstones. However, it may pose diagnostic difficulty due to an atypical presentation where the pain is localised to the left hypochondrium instead of the right in most cases [5]. Campos and Sipes published the first case report about performing a successful laparoscopic cholecystectomy (LC) on a patient with SIT in 1991 [6]. Laparoscopic cholecystectomy in these patients is different and technically more challenging due to the need for reorientation of the visio-motor skills to the left upper quadrant [5]. According to the systematic review and meta-analysis by Enciu et al. published in May 2022, more than 120 cases of situs inversus who underwent cholecystectomy were reported in the literature [7]. We found very few cases of morbidly obese patients with situs inversus totalis undergoing laparoscopic cholecystectomy after a literature search [8,9].

Herein, we report a morbidly obese patient with situs inversus totalis, who underwent a successful laparoscopic cholecystectomy in our institution.

## Case presentation

**Figure 1:**
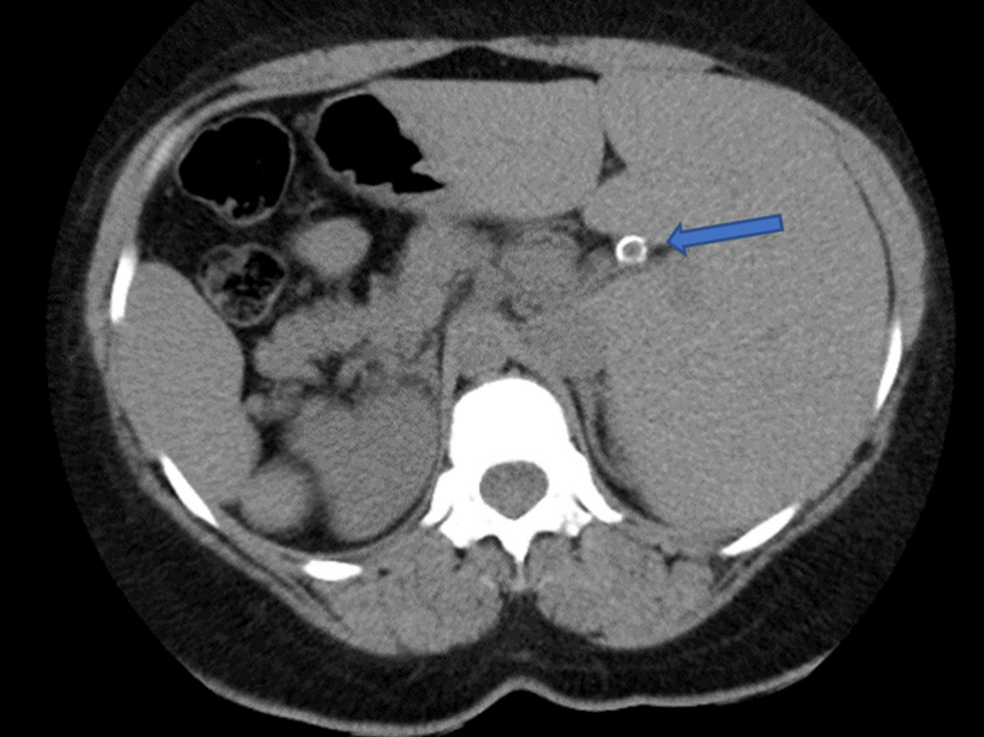
Plain CT of abdomen (axial view) showing right-sided spleen, left-sided liver with incidental finding of gallstones (Blue arrow). CT - computed tomography

A 38-year-old female, a known case of hypertension, bronchial asthma and morbid obesity with a body mass index (BMI) of 41, presented to the emergency department (ED) of our hospital with left upper abdominal and epigastric pain and nausea of two days duration, which was precipitated by a fatty meal. The patient reported similar pain on and off for the last year, and she was diagnosed to have gallbladder stones in the past but had deferred surgery. The patient stated that she had a condition called situs inversus totalis, which was recognized two years back when she had undergone a chest computed tomography (CT) with abdominal extension (Figure 1).

**Figure 2:**
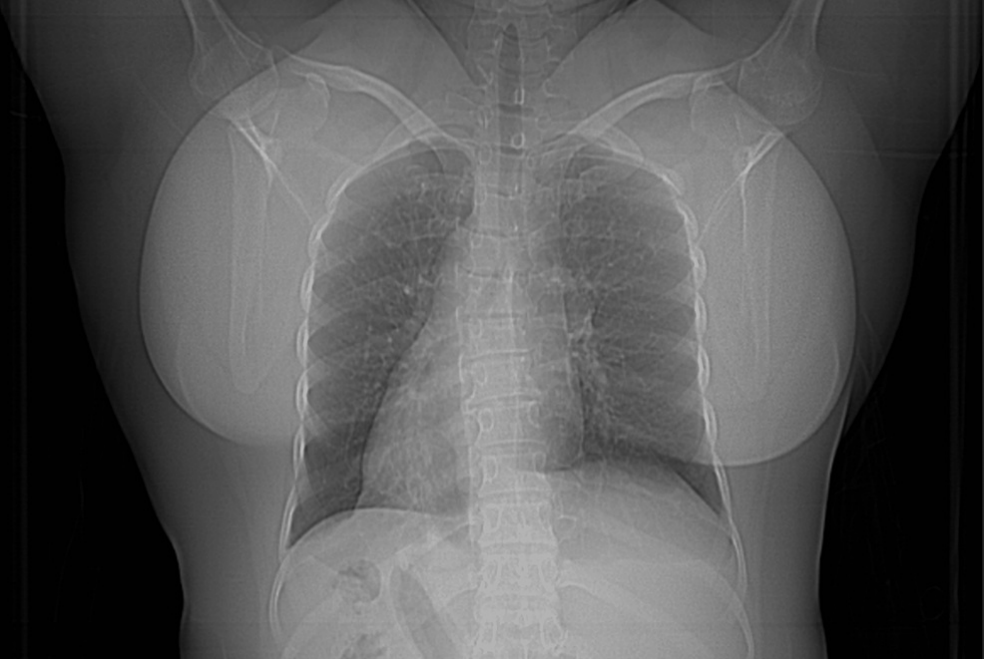
Chest X-Ray shows dextrocardia.

The patient was not in distress on examination with normal vital signs and oxygen saturation. Laboratory tests, like complete blood count, liver function tests, and basic metabolic panel, all were normal. Chest X-ray (Figure 2) showed dextrocardia without any obvious lung pathology.

**Figure 3:**
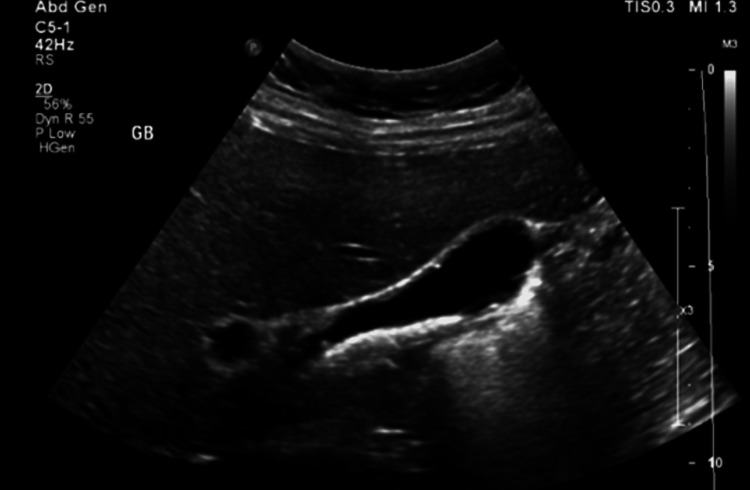
Ultrasonography of the gall bladder showing multiple gallstones.

Ultrasonography of the abdomen was performed, which showed multiple gallstones in the left-sided gall bladder without any signs of acute cholecystitis, like thickening of the gall bladder wall or pericholecystic fluid (Figure 3). Common bile duct was 4 mm and free of stones.

The patient was admitted to the surgical floor as a case of persistent biliary colic and booked for laparoscopic cholecystectomy on the next available slot after obtaining informed consent.

**Figure 4:**
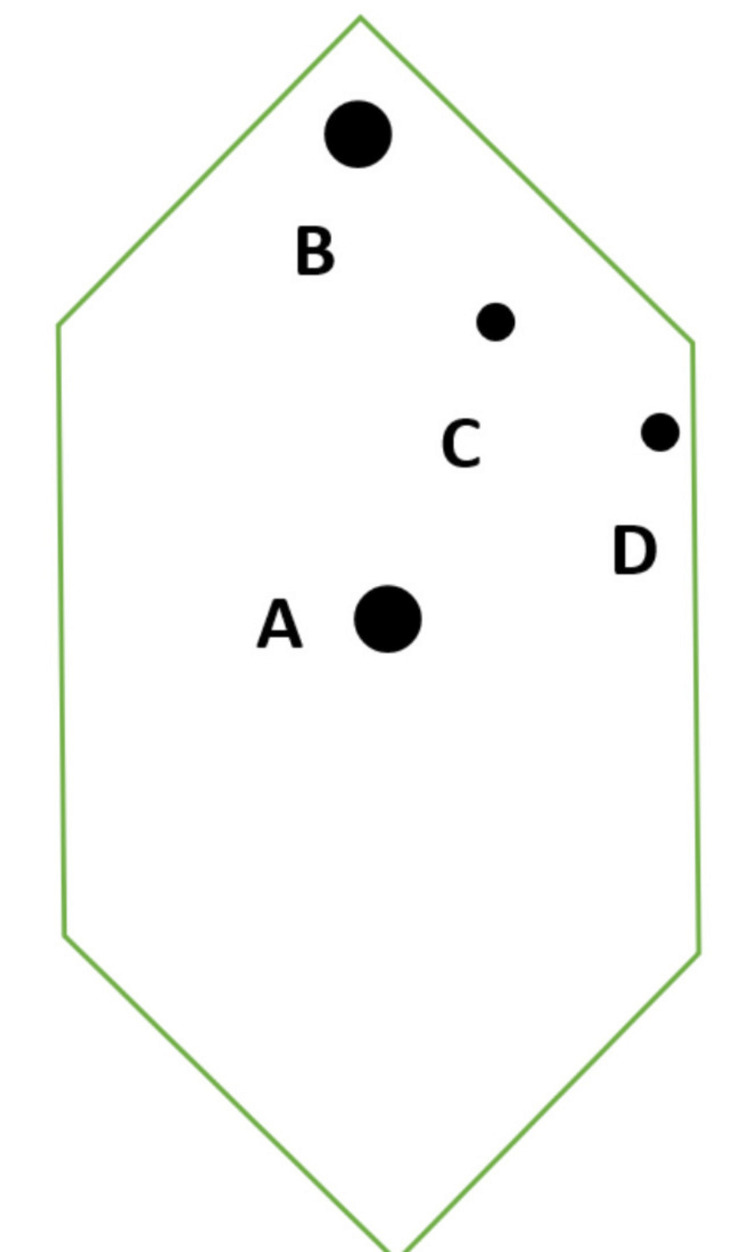
Port sites: A – umbilical 10 mm, B – subxiphoid 10 mm, C – midclavicular line 5 mm, D – anterior axillary line 5 mm

During surgery we used mirrored American LC technique with reversed mirror-image of routine laparoscopic cholecystectomy port sites (Figure 4).

We positioned the laparoscopic tower on the left side of the patient. The operating surgeon and camera operator were on the patient’s right side and the second assistant was positioned on the left. Upon laparoscopic exploration it was noted that all the abdominal viscera were reversed, but no other anomaly was observed. 

The procedure proved to be challenging, as expected, due to both the morbid obesity of the patient and the necessity of re-orientation to the left upper quadrant. Morbid obesity adds to the difficulty due to the thick abdominal wall precluding fine movements required at the tip of the laparoscopic instruments, especially if the left or non-dominant hand is used. The dissection of the hepatocystic triangle was the most demanding part of the procedure. Since the operating surgeon was right-handed, his hands were crossing during the procedure. To overcome this issue, the camera operator was assigned to help with the retraction of the neck of the gall bladder, while the operating surgeon carried on with the dissection of the critical view of safety by using mainly his right hand.

**Figure 5:**
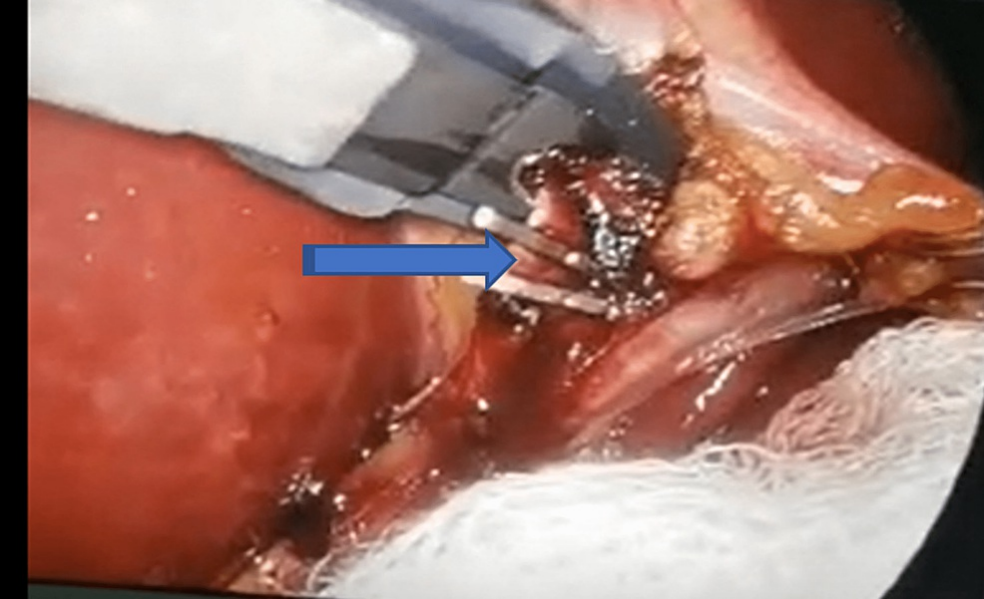
The cystic artery was clipped (Blue arrow)

**Figure 6:**
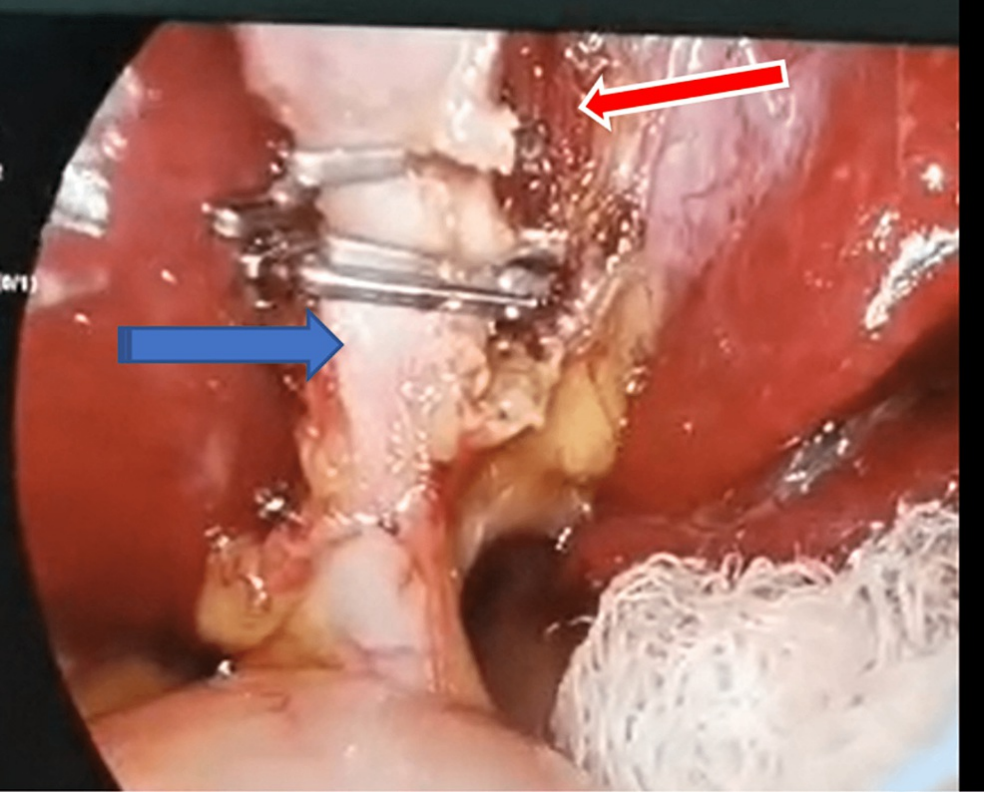
Clipped cystic duct (Blue arrow) and critical view of safety (Red arrow)

The cystic artery and duct were identified, skeletonized, clipped, and divided in the usual way (Figures 5, 6).

The surgery proceeded smoothly after dividing the cystic duct and artery. Dissection of the gallbladder from its bed was performed by using both right and left hands, the entire procedure took 75 minutes. The postoperative course was uneventful. We discharged the patient after two days of hospital stay, on the day following surgery in good condition.

## Discussion

Obesity is defined as a common, serious, and costly chronic disease of adults and children. Morbid obesity, on the other hand, tends to cause serious and devastating effects on many organs and systems in the body. Undoubtedly those patients suffering from morbid obesity are plagued with an increased risk of suffering from many chronic illnesses, like diabetes mellitis (DM), hypertension, joint disorders, cardiovascular conditions and even cancer [5]. Obesity is also one of the risk factors for cholelithiasis [5], the incidence of which is increasing worldwide. Therefore, surgeons are increasingly likely to encounter a growing number of obese patients who require a cholecystectomy for symptomatic cholelithiasis.

The number of LC in obese and morbidly obese patients keeps growing thanks to recent advances in minimally invasive techniques, rapidly developing technology and surgical equipment. There are many recent studies in the literature highlighting the safety of LC in morbidly obese and even super obese individuals, with equally good outcomes when compared to procedures done on patients with normal or near-nromal body mass index (BMI) [10]. One of the conclusions of these studies was that patients suffering from obesity or morbid obesity do not have higher complication or conversion rates, and even length of hospital stay (LOS) was not much different. The only difference seems to be operating time, which tends to be longer in procedures on patients with high or very high BMI.

Situs inversus totalis is a quite rare congenital condition where patients have reversed viscera, both thoracic and abdominal. Some patients with SIT may suffer from Kartagener’s syndrome or other abnormalities. Performing laparoscopic surgery on patients with SIT is undoubtedly challenging due to unfamiliar anatomy, the need for re-orientation of visio-motor skills and lack of cumulative experience on such cases. Despite the above-mentioned difficulties, it is safe to carry out LC on patients with SIT and it is considered a standard approach to the management of cholelithiasis even if the patient has reversed viscera [11].

Before the invention of minimally invasive surgery, cholelithiasis was managed by open cholecystectomy. There are about 40 reported open cholecystectomy cases in the literature that were carried out on patients with SIT. The introduction of minimally invasive surgery has undoubtedly revolutionized surgical practice. Very soon after the first successful laparoscopic cholecystectomy by Mouret in 1987, it became the gold standard in the management of gallbladder stones. After the first case report by Campos and Sipes in 1991, who described successful LC on a patient with SIT, more than 120 similar case reports have been published [7]. Interestingly, none of these cases reported any serious complications or have been converted to open cholecystectomy, which is quite surprising, considering how challenging the laparoscopic procedure can be on those individuals. The reason for this fact could be multifactorial; first, it is presumable that most or all laparoscopic surgeries on patients with SIT have been performed by expert laparoscopic surgeons with excellent manual dexterity. Second, mostly successful surgeries tend to be reported by authors. The fact that SIT is a rare condition and the patient with SIT presenting with cholelithiasis is even more rare, such patients are usually thoroughly investigated, imaged, surgical procedures are well planned and utmost care is taken not to miss any possible anomaly or comorbidity, which may result in unusual excellent outcome in these patients. LC has been proven to be a safe procedure, and not contraindicated at all in patients with SIT [12].

The reported cases of laparoscopic cholecystectomy in morbidly obese patients with SIT are extremely rare. We found very few case reports describing laparoscopic cholecystectomy in morbidly obese patients with SIT after a literature search [8,9].

Our patient was morbidly obese and a known case of SIT, who presented with left upper quadrant and epigastric pain, so diagnosis was not a challenge for us. However, it is noteworthy to mention that if the patient is not a known case of SIT, presentation, symptoms and signs of many conditions, including cholelithiasis, may pose a diagnostic challenge to clinicians. Imaging, like total abdominal sonography and computed tomography, usually reveals what's hidden, but some patients may suffer for years before being diagnosed, especially in underprivileged countries where even abdominal sonography could be a subject of availability [13].

In about 60% of cases of acute cholecystitis in patients with SIT, the main complaint was epigastric and left hypochondrium pain according to previously published studies, whereas about 30% of patients suffered epigastric pain alone. Interestingly, in about 10% of cases, the pain was localised to the right hypochondrium. Some authors suggested the possibility of lack of sharing of the central nervous system in the reversal of organs in such cases [14].

The American mirror technique seems to be the most prevalent technique among surgeons who have performed laparoscopic cholecystectomy on patients with SIT [7]. Some authors mentioned the difficulties related to changing the operating surgeon’s position, port site insertion, using opposite hands for handling the instruments and the opposite foot to operate the foot pedal. All these challenges have caused almost doubling of the operative time.

Performing LC on morbidly obese patients with SIT is more challenging due to the thick abdominal wall and difficult access to the gallbladder neck caused by a large amount of intraabdominal fat. A thick abdominal wall interferes with fine movements at the tip of laparoscopic instruments, especially if the non-dominant hand is used.

LC in patients with SIT is easier for left-handed surgeons according to some authors. The fastest technique for right-handed surgeons seems to be the American mirror technique and some modifications of the port placement can facilitate it [7].

In our case, we used the commonly employed American mirror technique using four ports. We inserted two 10-mm ports: one in supraumbilical position, the other one in subxiphoid area, to the left of the falciform ligament, while other 5 mm ports were inserted on the left side, one on the anterior axillary line, the other on subclavicular line, below the left costal margin. Skeletonizing the structures in Calot's triangle consumed extra time and proved to be more difficult than in patients with a normally situated gallbladder. Dissection of the gallbladder from its bed and removal of the specimen was not so difficult, considering that the procedure was carried out by an experienced surgeon with good manual dexterity. The procedure was successfully accomplished without significant prolongation of the operative time; the entire procedure took 75 minutes, while the mean operative time was reported to be 80 minutes (range: 15 - 281 minutes) in one study [15].

## Conclusions

Laparoscopic cholecystectomy in patients with SIT is feasible and safe but more challenging, especially in morbidly obese patients due to the thick abdominal wall interfering with fine movements at the tip of laparoscopic instruments, especially during use of the non-dominant hand. It is considered a daunting procedure and requires technique modulations compared to the standard laparoscopic cholecystectomy. The related anatomical abnormalities of other structures too should be excluded preoperatively to avoid complications.

In patients with SIT, laparoscopic cholecystectomy should be done by very experienced laparoscopic surgeons with impeccable dexterity to reduce the likelihood of complications. In our opinion, being right- or left-handed has no significant bearing on the outcome, as indicated by the great majority of the reported cases in the literature being done by right-handed surgeons. It is preferable to have two assistants for LC in patients with SIT, with one retracting the gallbladder and the other holding the camera. This allows the operating surgeon to use his/her dominant hand during the dissection of Calot’s triangle, which is the most critical part of the procedure.
